# Using explainable machine learning and eye-tracking for diagnosing autism spectrum and developmental language disorders in social attention tasks

**DOI:** 10.3389/fnins.2025.1558621

**Published:** 2025-06-24

**Authors:** Adoración Antolí, Francisco Javier Rodriguez-Lozano, José Juan Cañas, Julia Vacas, Fátima Cuadrado, Araceli Sánchez-Raya, Carolina Pérez-Dueñas, Juan Carlos Gámez-Granados

**Affiliations:** ^1^Department of Psychology, University of Córdoba, Córdoba, Spain; ^2^Maimónides Biomedical Research Institute of Córdoba, Córdoba, Spain; ^3^Department of Electronic and Computer Engineering, Higher Polytechnic School, University of Córdoba., Córdoba, Spain; ^4^Mind, Brain and Behavioral Research Center, University of Granada, Granada, Spain

**Keywords:** explainable machine learning, autism spectrum disorder, developmental language disorder, eye-tracking, differential diagnosis, computer-aided diagnosis

## Abstract

**Background:**

Eye-tracking technology has proven to be a valuable tool in detecting visual scanning patterns associated with autism spectrum disorder (ASD). Its advantages in easily obtaining reliable measures of social attention could help overcome many of the current challenges in the assessment of neurodevelopmental disorders. However, the clinical use of this technology has not yet been established. Two key challenges must be addressed: the difficulty in reliably distinguishing between disorders with overlapping features, and the efficient management of eye-tracking data to yield clinically meaningful outcomes.

**Purpose:**

The aim of this study is to apply explainable machine learning (XML) algorithms to eye-tracking data from social attention tasks involving children with ASD, developmental language disorder (DLD), and typical development (TD), in order to assess classification accuracy and identify the variables that best differentiate between groups.

**Methods:**

Ninety-three children participated in a visual preference task that paired social and non-social stimuli, specifically designed to capture features characteristic of ASD. Participants were distributed across three groups: ASD (*n* = 24), DLD (*n* = 25), and TD (*n* = 44). Eye-tracking data were used to generate four datasets, which were then analyzed using XML algorithms to evaluate the accuracy of group classification across all possible combinations.

**Results:**

The model achieved an F1-score of 0.912 in distinguishing DLD from TD, 0.86 for ASD vs. TD, and 0.88 for the combined ASD+DLD group vs. TD. Performance was moderate for ASD vs. DLD, with an F1-score of 0.63. The most informative areas of interest were those broadly grouping social and non-social stimuli, while more specific variables did not improve classification accuracy. Naive Bayes and Logistic Model Trees (LMT) emerged as the most effective algorithms in this study. The resulting model enabled the identification of potential disorder-specific markers, such as the mean duration of visits to objects.

**Conclusion:**

These findings highlight the potential of applying XML techniques to eye-tracking data collected through tasks designed to capture features characteristic of neurodevelopmental conditions. They also underscore the clinical relevance of such approaches for identifying the variables and parameters that differentiate between disorders.

## 1 Introduction

### 1.1 Neurodevelopmental disorder characteristics

Autism spectrum disorder (ASD) is a neurodevelopmental disorder (NDD) defined by the presence of persistent deficits in social interaction and communication across multiple contexts, and repetitive and restrictive patterns of behaviors, interests, and activities (American Psychiatric Association [APA], 2022). An early diagnosis is essential to apply efficient interventions, but, at the same time, it is a challenge at 2 or 3 years of age because the behavioral repertoire is still very restricted, above all in oral language. There is another NDD that shares communicative impairments with ASD – developmental language disorder (DLD) (American Psychiatric Association [APA], 2022), also known as specific language impairment or language disorder. DLD is a developmental condition characterized by severe and persistent deficits in the acquisition or use of language, with a great impact on individuals’ daily functioning (American Psychiatric Association [APA], 2022; Bishop et al., 2016, 2017; World Health Organization [WHO], 2019). DLD affects communication and secondarily social skills and emotional development (Aguilar-Mediavilla et al., 2022). Communicative impairments are a characteristic shared between DLD and ASD, and it makes the differential diagnosis difficult at early ages (Bishop, 2010; Vacas et al., 2021a).

The most recent estimated prevalence of DLD ranges from 7.58% in children aged 5–6 years in the United Kingdom (Norbury et al., 2016), to 6.4% in 10-year-old children in Australia (Calder et al., 2022), and 8.5% in Mandarin-speaking children aged 5–6 years (Wu et al., 2023). This prevalence is nearly seven times higher than that of ASD. While the global prevalence of ASD has been estimated at approximately 1% (Brugha et al., 2012; Zeidan et al., 2022), considerable regional variability has been reported, with rates reaching 2.23% in the USA (Maenner et al., 2023), 0.36% in Asia (Qiu et al., 2020), and 0.7% in China (Zhou et al., 2020). These variations likely reflect differences in diagnostic criteria, sampling methods, and sociocultural factors. Despite these prevalence rates, research on DLD remains limited (McGregor, 2020), and even less on ASD and DLD jointly. However, both disorders have different severities and prognoses (McGregor, 2020) and require applying different supports for children and families. All this makes it necessary to have instruments for early differential diagnostics (Bishop et al., 2016; Georgiou and Spanoudis, 2021; Rice, 2016; Vacas et al., 2021b,2022a; Weismer, 2013).

ASD and DLD have been considered different conditions, with language difficulties well-differentiated in each disorder. However, the wide heterogeneity of both profiles, in language, autistic traits, and social behavior (Bishop, 2000; Bishop and Norbury, 2002; Conti-Ramsden et al., 2006), has raised the issue of the unclear boundaries between these disorders. In this context, a differential visual scanning pattern with social and non-social objects may help to distinguish between conditions (Vacas et al., 2021c,^2024^).

### 1.2 Eye-tracking for neurodevelopmental disorders screening

Using eye movement-based markers as screening tests for ASD is yielding highly encouraging outcomes. This may be partly due to the fact that eye-tracking provides a direct and sensitive measure of gaze behavior during visual stimulus processing (Mastergeorge et al., 2021). Thus, most studies in the field have aimed to distinguish between individuals with typical development and those with an ASD diagnosis or risk, while to a lesser extent incorporating comparisons with other clinical groups (e.g., DLD) to establish a differential diagnosis. However, the main challenge in clinical practice arises from the early differential diagnosis of disorders that share similar characteristics, like with ASD and DLD. Eye-tracking has been used in research on NDDs, especially with ASD but less with DLD (Vacas et al., 2022a).

Another challenge in employing eye-tracking methodology for NDD screening is the use of diverse tasks and measurements of a wide range of eye-tracking parameters and variables, significantly complicating comparisons across different studies. Presented stimuli can be static or dynamic, singular or multiple simultaneously, social or objects, displaying emotions or not, etc.; and the participant’s elicited response may vary from passive observation to more complex engagement such as emotion recognition, situational interpretation, or even interaction (Mastergeorge et al., 2021; Setien-Ramos et al., 2023). These procedural differences have implications for results, interpretations, and the selection of the most suitable procedures and metrics as specific markers for distinct NDDs, a task yet to be fully addressed (Vacas et al., 2022a). Studies that facilitate comparisons across different disorders can assist in identifying eye-tracking variables, stimuli, and tasks that best differentiate between these disorders and elucidate the affected processes underlying such visual behavior (Vacas et al., 2024).

### 1.3 Machine learning for neurodevelopmental disorders screening

Machine learning (ML) techniques have emerged as valuable tools in research on the assessment and diagnosis of NDD (Moreau et al., 2023); its main advantage over traditional methods is its ability to process large volumes of heterogeneous data and detect latent patterns that are not evident through conventional analysis. Nevertheless, their direct application in clinical practice still remains limited. Recent studies have implemented ML for the analysis of data from the Autism Diagnostic Observation Schedule (ADOS), achieving accurate differentiation between individuals with ASD and those with typical development (TD), with AUC (area under the curve) values of 0.95 for Module 3 and 0.93 for Module 2 (Levy et al., 2017), and accuracy values of 0.89 for children/younger adolescents (Kamp-Becker et al., 2021). Similarly, ML techniques have been applied to distinguish between ASD and attention-deficit/hyperactivity disorder (ADHD), using data from the Social Responsiveness Scale (SRS), resulting in significant improvements in classification (Duda et al., 2016). Nevertheless, these approaches do not eliminate the need for neuropsychological testing, which requires a substantial time investment and specialized training. For this reason, it is essential to continue gathering data to apply ML algorithms, with the aim of progressively reducing reliance on neuropsychological testing while maintaining or even improving the accuracy of profile classification.

In addition to the use of clinical instrument data, ML applications have been explored with data obtained from electroencephalography and magnetic resonance imaging, optimizing feature selection and diagnostic classification (Arbabshirani et al., 2017; Heinsfeld et al., 2017; Levy et al., 2017; Mazumdar et al., 2021; Rahman et al., 2020). These methodologies have shown potential for identifying specific biomarkers associated with ASD, although their clinical applicability is still in preliminary stages. Less common applications include the use of data from linguistic productions (Kato et al., 2024; Parikh et al., 2019) and genetic analyses (Nahas et al., 2024).

The integration of multimodal data -including behavioral, clinical, neuroimaging, and genetic features- has become increasingly common in recent years to improve the screening and diagnosis of NDDs (Bone et al., 2016; Levy et al., 2017; Lombardo et al., 2019; Wang et al., 2024).

Given the potential of ML to handle large volumes of data, combining ML with eye-tracking methodology is particularly valuable, especially for extracting complex visual exploration patterns related to specific NDDs. For example, recent studies have demonstrated that gaze behavior, when analyzed with ML techniques, can reveal complex, nonlinear markers associated with autism spectrum disorder and cognitive development (Wang et al., 2023; Wei et al., 2023; Zhou et al., 2023). This approach can enhance diagnostic accuracy and screening (Kollias et al., 2021). These studies, along with broader reviews such as Moreau et al. (2023), highlight the ongoing shift toward multimodal, dynamic, and interpretable ML applications in neurodevelopmental screening.

Typically, the use of ML in addressing NDDs has focused on comparing ASD with TD, while only a few studies have compared different NDD groups with each other. This aspect increases the risk of bias in studies by overestimating the accuracy of assessment or classification, as it reduces the symptom overlap that exists between different disorders (Whiting et al., 2011). Additionally, a differential diagnosis of NDDs that share similar symptoms is often very challenging, especially at early ages when the range of behaviors is limited. Again, gaps are observed in the characterization, early identification, and differentiation of NDDs, making it necessary to continue research to meet the demands of clinical practice.

Within ML technology, and to leverage the benefits of using ML in NDDs, different types of algorithms can be employed. In this study, given the nature of the problem, we focused on classification-oriented algorithms. In this way, a predictive model was generated to classify the participants of the study into different diagnostic groups (TD, ASD, and DLD). Besides looking for a good model, this work pursued the objective of studying which eye-tracking group of variables (dataset) better differentiated participants based on their diagnosis. Datasets were designed with more or fewer variables, and with different types of information about ocular behavior (parameters) and type of stimulus (objects or faces, emotions, specific areas of the face such as the mouth and eyes). These datasets were specifically constructed to capture differences in visual attention to social and non-social stimuli, considering parameters and stimulus identified in the literature as sensitive to variations in gaze behavior across NDDs (Jónsdóttir et al., 2023; Polzer et al., 2024; Sasson and Touchstone, 2014), and metrics such as fixation duration, fixation count, and latency to first fixation have been shown to effectively distinguish between individuals with ASD, DLD and TD (Setien-Ramos et al., 2023; Vacas et al., 2024).

### 1.4 Algorithms of explainable artificial intelligent

In mental health, different artificial intelligence (AI) models, such as explainable AI (XAI) and “black box” approaches, are being used. These “black box” approaches often involve deep learning models, such as multilayer neural networks, which have been applied to neurodevelopmental screening using neuroimaging (Heinsfeld et al., 2017), speech analysis (Parikh et al., 2019) and health administrative data (Dick et al., 2025). These models do not provide direct insight into how or why a specific classification is made. Although both types of algorithms aim to maximize classification accuracy, XAI models provide a transparent interpretation of the variables that influence model decisions (Joyce et al., 2023; Sangwan, 2024). This capability is crucial in neuropsychology, as it allows for the identification of which behavioral variables or neuropsychological responses are modulating the models predictions. In this study, XAI algorithms were used to comprehensively analyze the parameters that differentiated between various clinical groups, fostering a better understanding of the underlying processes and their potential application in clinical contexts. Especially interesting was the possibility to test models that may be interpretable by experts.

The XAI-based algorithms have different approaches; some are based on popular decision trees, rule sets, or probabilistic functions, etc. In this study, inherently interpretable algorithms such as Naive Bayes, RIPPER, One Rule, PART, C4.5 (J48), and Logistic Model Tree (LMT) were selected due to their ability to provide directly understandable explanations. Unlike black-box models, whose decision-making processes are opaque and require post hoc explanation tools such as SHAP (SHapley Additive exPlanations; Lundberg and Lee, 2017) or LIME (Local Interpretable Model-agnostic Explanations; Ribeiro et al., 2016) to decompose their predictions, interpretable models present decision rules, tree structures, or probabilistic relationships that can be easily understood and validated by experts (Molnar, 2022). While black-box models often achieve higher accuracy in complex scenarios, their interpretability is partial and depends on the faithfulness of the generated explanations, which can be problematic in clinical or educational contexts where transparency and traceability of decisions are essential (Hassija et al., 2023).

Furthermore, we included two groups of disorders (ASD and DLD), as well as a TD group, with the objective of analyzing classification accuracy for different combinations of disorders. In addition, the eye-tracking protocol used for data collection was designed while considering the specific characteristics of social attention in individuals with ASD.

Thus, the main objective of this study was to explore and select the most efficient XML algorithms to distinguish between TD, ASD, and DLD samples, using eye-tracking data in order to assist clinicians in making decisions. To pursue this general goal, we pursued three specific objectives: (1) study which dataset (with more or fewer variables) is the most useful for generating explainable classification models; (2) determine which dataset better differentiates participants based on their diagnosis; and (3) analyse which ML model generates the best results for each comparison, identifying the characteristics (eye-tracking metrics) that are determinant in discriminating between groups of participants, and ensuring these models are interpretable by experts.

## 2 Materials and methods

This study involved two phases: (1) sample recruitment, task performance, and data transformation, and (2) ML algorithm-testing.

### 2.1 Phase 1: sample recruitment, task performance, and data transformation

#### 2.1.1 Participants

The sample in this study consisted of 93 young children aged between 32 and 74 months (*M* = 53.51; *SD* = 10.6), who were divided into three groups according to their diagnosis: (1) TD group (*n* = 44), (2) ASD group (*n* = 24), and (3) DLD group (*n* = 25). Both clinical groups (ASD and DLD) were recruited from centers of early childhood intervention in province of Córdoba (Spain). Inclusion criteria for these groups comprised: (1) the adscription to an early childhood intervention center; (2) a formal diagnosis of ASD or DLD assessed by a licensed, experienced team of clinicians, following the guidelines of the international diagnostic manuals (DSM-5, American Psychiatric Association [APA], 2022; and ICD-11, World Health Organization [WHO], 2019) and the protocol of the Infant Mental Health program at a community mental health service; and 3) the absence of any comorbid condition, which was confirmed by the professionals from the early childhood intervention center. Conversely, TD participants were recruited from a public school in the same province. Inclusion criteria in this case comprised: (1) the absence of any developmental condition or formal diagnosis of NDD either now or in the past, and (2) the chronological age matching with both clinical groups.

Participants’ features appear in Table 1. We defined our groups in terms of age, gender, basic attention (percentage of fixations during the eye-tracking task, which is an indicator of the ability of participants to fulfill the requirements to complete the task), and the level of receptive vocabulary, measured with the Peabody Picture Vocabulary Test-Third Edition (PPVT-III) (Dunn and Dunn, 1997).

**TABLE 1:** Sample features.

	TD (*n* = 44)	ASD (*n* = 24)	DLD (*n* = *25*)		
	*M* (*SD*)	*M* (*SD*)	*M* (*SD*)	*F*_(2, 90)_	*P*
Age (months)	53.23 (10.04)	53.50 (10.35)	54 (12.14)	.041	0.959
Basic attention	87.09 (11.62)	73.50 (12.66)	73.08 (15.21)	13.23	<0.01**
PPVT-III standard score	108.75 (10.82)	98.65 (12.05)	86.57 (18.90)	19.56	<0.01**
	*N*	*n*	*N*	*χ^2^*	*P*
Gender	22/22	22/2	23/2	20.15	<0.01**

Age, basic attention, and PPVT-III Standard Score variables were tested with ANOVA. Gender was analyzed with chi-square testing. Significance levels: ***p < 0.001; **p < 0.01; and *p < 0.05.

As Table 1 shows, the groups did not differ in age, but they did regarding the other variables. *Post-hoc* tests revealed that the TD group displayed more basic attention compared with both clinical groups (*p* < 0.001, in both cases) and all groups showed differences regarding the PPVT-III Standard Score (TD-ASD: *p* = 0.02; TD-DLD: *p* < 0.001; ASD–DLD: *p* = 0.016). Finally, the groups also differed in terms of gender, with the TD group having an equal proportion of boys and girls, while the clinical groups only included two girls each. This difference in sex rate was in line with most reports, indicating that these disorders are more prevalent in male than female populations (American Psychiatric Association [APA], 2022; Pérez-Crespo et al., 2019).

This study was approved by the Research Ethics Committee of Córdoba (Spain). Following the principles of the Declaration of Helsinki drafted by the WHO, families of all participants were informed of the purpose of the study and the assessment procedures, and they were asked to give written informed consent to authorize their children to take part in this study.

#### 2.1.2 Apparatus and stimuli

The Tobii X2-30 remote eye-tracker (Tobii Technology AB, Stockholm, Sweden) was used to perform the eye-tracking function at a sampling rate of 30 Hz with a spatial accuracy of 36°. The device belongs to the category of screen-based eye-trackers. It was conveniently placed at the bottom of a 15” laptop screen.

**FIGURE 1:**
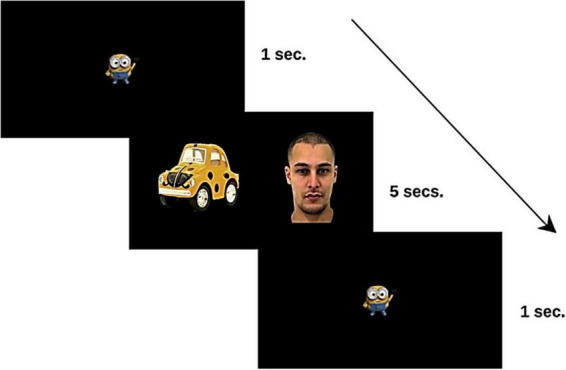
Sequence of stimuli presented in each trial to the study participants. The facial images were taken from the Amsterdam Dynamic Facial Expression Set (ADFES; Van der Schalk et al., 2011).

**FIGURE 2:**
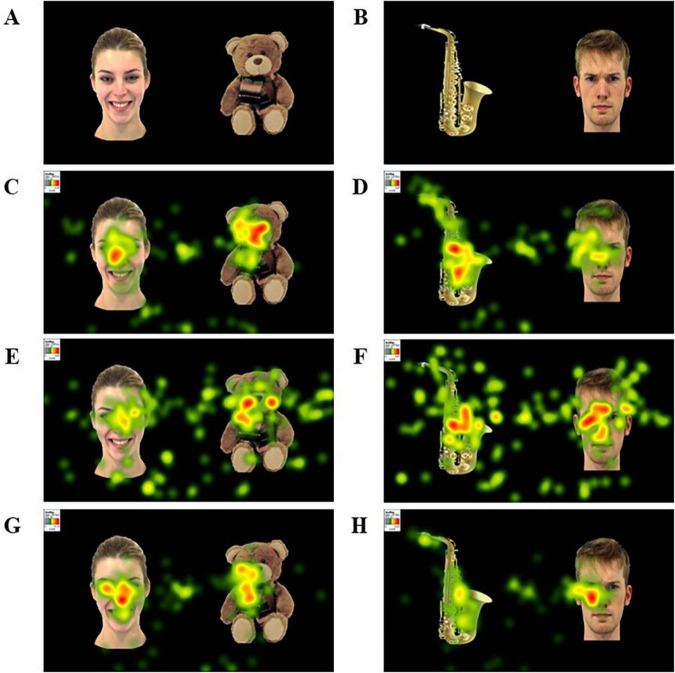
Examples of stimuli and heatmaps for the ASD, DLD, and TD groups. **(A)** Example stimulus pairing a happy face and a CIO. **(B)** Example stimulus pairing an angry face and a non-CIO. **(C,D)** Heatmaps of the ASD group performance. **(E,F)** Heatmaps of the DLD group performance. **(G,H)** Heatmaps of the TD group performance. The facial images were taken from the Amsterdam Dynamic Facial Expression Set (ADFES; Van der Schalk et al., 2011).

Stimuli were presented with the Tobii Studio software and a 9-point calibration, with an animated stimulus as target. Stimuli were designed following the eye-tracking paired preferences paradigm, which consists of pairing social and non-social images to assess visual attention patterns for faces and objects used in the eye-gaze literature (Sasson and Touchstone, 2014; Vacas et al., 2021c). Faces displaying three different emotions (happiness, anger, and neutral) were paired with two types of objects [related to autistic circumscribed interests (CIOs) and unrelated to them (non-CIOs)] in each trial. Six experimental conditions were repeated six times, using different facial identities (36 trials in total, see Figure 1). The gender of the faces and their location on the screen were counterbalanced to avoid the potential effects of both variables. The facial images were taken from the Amsterdam Dynamic Facial Expression Set (ADFES; Van der Schalk et al., 2011) and were paired with images of objects (CIOs and non-CIOs). These images were taken either from the Pixabay website,^1^ free of copyright under the Creative Commons CC0 license, or from our own creation. Selection criteria for CIOs were based on previous studies (Sasson et al., 2008; Sasson and Touchstone, 2014; South et al., 2005). CIOs belonged to the categories of blocks, means of transport, animals, puzzles, and toys, while non-CIOs were clothes, plants, musical instruments, school materials, tools, and furniture (see Figure 2).

#### 2.1.3 Task performance procedure

The participants were assessed in their natural environment (their referential center of early childhood intervention for participants with ADS/DLD, and their schools for TD participants), using a quiet room without distraction. First, participants were seated at a deemed distance of 60 cm from the laptop with the eye-tracker, which displayed the paired preference task. They were given no other instruction but to look at the screen. After calibration, the task consisted of visualizing the set of 36 slides showing one face and one object for a total time of 3.6 min (5 s per slide). Prior to the presentation of each slide, participants viewed an animated fixation point for 1 s to drive their attention to the center of the screen (see Figure 1). After the eye-tracking task, receptive vocabulary was assessed with the PPTV-III (Dunn and Dunn, 1997). After completing the assessment session, all participants received a gift as a reward.

#### 2.1.4 Data transformation

This study aimed to identify the characteristics (eye-tracking metrics) that are determinant in discriminating between groups of children. To do so, the eye-tracking software allows one to create areas of interest (AOIs) to provide data only about the most relevant elements of the stimuli. The selection of AOIs was guided by aspects that have been shown to differ in individuals with ASD, such as emotion processing (Polzer et al., 2024; Vacas et al., 2022b), facial scanning patterns—particularly attention to the eyes and mouth—(Jónsdóttir et al., 2023); and the type of object especially whether it is associated with the circumscribed interests commonly observed in autism (Sasson and Touchstone, 2014; Vacas et al., 2021c). In this study, we defined five prior AOIs: (1) happy faces, (2) angry faces, (3) neutral faces, (4) CIOs, and (5) non-CIOs. For each emotional face, we designed two additional AOIs (one for the eyes and another one for the mouth), and we also created two extra AOIs for the total of faces and objects. This resulted in 13 AOIs (happy face, happy eyes, happy mouth, angry face, angry eyes, angry mouth, neutral face, neutral eyes, neutral mouth, CIO, non-CIO, total faces, total objects).

On the other hand, Tobii Studio provides information about 10 eye-tracking raw metrics: (1) time to first fixation (TFF); (2) fixations before (FB); (3) first fixation duration (FFD); (4) fixation duration (FD); (5) total fixation duration (TFD); (6) fixation count (FC); (7) visit duration (VD); (8) total visit duration (TVD); (9) visit count (VC); and (10) percentage fixated (PF). Additionally, we created two extra eye-tracking metrics to assess the proportion of fixation duration on each AOI (PFD) and the meantime per visit (TPV) by dividing TVD/VC. This resulted in 12 eye-tracking metrics. Figure 2 presents a heatmap displaying the FD metric for a specific stimulus across different participant groups.

The combination of the eye-tracking metrics for each AOI yielded a total of 156 variables. To determine which of these variables are truly necessary to achieve the highest classification accuracy in distinguishing between groups (ADS, DLD, and TD), they were organized into four datasets based on their level of specificity, to be tested with the algorithms. (1) “basic variables,” comprising all metrics regarding the AOIs of total faces and total objects (24 variables); (2) “prior variables,” which involved all metrics regarding the AOIs of happy faces, angry faces, neutral faces, CIOs, and non-CIOs (60 variables); (3) “secondary variables,” including all metrics regarding the AOIs of eyes and mouth in each emotional face (72 variables); and (4) “full variables,” with the 156 eye-tracking variables plus two sociodemographic variables (gender and age) (158 variables).

### 2.2 Phase 2: ML algorithm testing

Following the objective of obtaining results that allow for the interpretation of the obtained model’s outcomes, we selected and tested the following XML algorithms:

*Naive Bayes*: Naive Bayes (Domingos and Pazzani, 1997) is a probabilistic method, which, as its name suggests, is based on the calculation of the Bayes theorem, which calculates the a posteriori probability, that is, the membership of new patterns to a class, based exclusively on previous observations. Thus, the probability calculated with Equation 1, and therefore the label and the corresponding class of a new data, is given by the class with maximum probability:


(1) $$P\,\left(y_{n}|a_{1}\,a_{j}\right)=P\,\left(y_{n}\right)\cdot \prod_{i=1}^{j}P\,\left(a_{i}|y_{n}\right)$$

Let *P*(*y*_*n*_|*a*_1_*a*_*j*_) be the probability of belonging to the class, considering all its attributes {*a*_1_…*a*_*j*_}; *n*: number of classes; *j*: total number of attributes; *P*(*y*_*n*_), the probability of an instance to belong to the class; $\prod_{i=1}^{j}P\,\left(a_{i}|y_{n}\right),$the conditional probability of *a*_*i*_attribute, given the class *y_n_*.

*Ripper*: Ripper stands for repeated incremental pruning to produce error reduction, or in short, the RIPPER (Cohen, 1995) algorithm, which is an iterative method based on the generation of a model based on interpretable rules. RIPPER is an algorithm designed to be able to find a specific set of rules to classify with higher accuracy the classes that have a smaller number of samples. The general operation consists of iterating each class, starting from the minority to the majority, and for each of them dividing the training set into two, one pruning and one growth. Each rule grows in conditions until there are no more samples of a class in the growth set or the generated rules that provide an error greater than 50%. After this, the rules are optimized by choosing those that have a minimum value in the decision length (DL) metric. Once the set of rules has been obtained, those that increase the DL metric are eliminated (Equation 2).


(2) D⁢L⁢(H,D)=D⁢L⁢(H)+D⁢L⁢(D|H)

where *H* is the set of rules, *D* is the training dataset, *DL(H)* represents the length of the encoded rule set, and *DL(D| H)* is the cost of encoding the misclassified examples.

*One Rule:* One Rule (OneR) (Holte, 1993) is an algorithm that generates concrete rules for a given feature. It is an extremely simplified algorithm in which all rules will have only one single feature. Therefore, the set of rules is defined by rules in which all have in common a single attribute that discriminates between classes. Because of the way it works, it is necessary that the characteristics that identify the different subjects are discretized or categorized.

To generate the rules around a single feature, the algorithm iterates over all the features in the training set and for each value of each of the features the specific samples that have that feature, and its associated class label is obtained. Once all the labels have been obtained, the majority class is considered to be the one in which the label count for that class is the maximum. The remaining samples that do not correspond to that feature are considered errors, so that for each feature, the total error for a given value is the sum of these errors. After calculating all the errors, the feature that best separates the data into the different classes is the one for which the sum of the errors of its possible values is minimum (Equation 3).


(3) $$\hat{y}\left(x\right)=\arg maxP\left(c|x_{j}=v\right)$$

where:


$$x_{j}\,\mathrm{is}\,\mathrm{the}\,\mathrm{variable}\,\mathrm{selected}\,\mathrm{as}\,\mathrm{the}\,\mathrm{best}\,\mathrm{according}\,\mathrm{to}\,\mathrm{OneR},$$

$$v\,\mathrm{is}\,\mathrm{the}\,\mathrm{value}\,\mathrm{of}\,x_{j}\,\mathrm{in}\,\mathrm{the}\,\mathrm{example}\,x,$$

c⁢is⁢the⁢class,

$$P\left(c|x_{j}=v\right)\mathrm{is}\mathrm{the}\mathrm{proportion}\mathrm{of}\mathrm{class}c$$

observed⁢in⁢the⁢training⁢set⁢for⁢that⁢value⁢v.

*Partial Decision Trees*: The partial decision tree (PART) algorithm (Frank and Witten, 1998) is a method that, like the previously described RIPPER, is a rule-induction method. Like RIPPER, it follows the “divide and conquer philosophy” to generate rules, with the difference that the rules are organized hierarchically into a tree structure. This method, unlike C4.5 and RIPPER, avoids complex optimization steps or adjustments to modify individual rules in the rule set. To limit the deep growth of the tree (depth levels nested in the rules), a pruning step is performed to simplify the generated tree.

This method uses (like C4.5) the entropy metric to perform the divisions of the tree branches. In this way, the set of examples is divided into subsets and the sets are recursively subdivided into branches until only leaf nodes remain. Once the level of leaf nodes has been reached, it is checked whether the error that the subset of the tree is greater or smaller than that estimated for the node. If it is less, the subtree is simplified by directly generating a leaf node that replaces the subtree (Equation 4).

Let *R* = {*r*_1_,*r*_2_,…,*r*_*m*_}be the set of generated rules. Each rule the following form:


(4) $$r_{k}\,\left(x\right)=\left\{ \begin{array}{cc} c_{k}, & \text{if}\,C\,o\,n\,d_{k}\,\left(x\right)=t\,r\,u\,e,\\ \mathrm{not}\,\mathrm{applicable}, & \text{if}\,C\,o\,n\,d_{k}\,\left(x\right)=f\,a\,l\,s\,e, \end{array}\right.$$

where: *c_k_*is the class predicted by rule *r_k_*, *Cond*_*k*_(*x*) is the logical condition over the variables (e.g., *x*_1_ = 5∧*x*_3_ = *A*). The final prediction is:


$$\hat{y}\,\left(x\right)=c_{j},j=m\,i\,n\,\left\{k|C\,o\,n\,d_{k}\,\left(x\right)=t\,r\,u\,e\right\}$$

*C4.5:* C4.5 (Quinlan, 1993) is a method based on discrimination between classes that generates a tree with two types of elements – decision nodes and leaves. The decision nodes are the separations that depend on the values of a certain attribute. The leaves represent the label that corresponds to the data after following the path traced by the different decision nodes. In this way, a tree is generated where the decision nodes are generated, using the gain of information provided by a particular attribute, this being the one that best divides the data set in each decision. As in the OneR method, the values corresponding to each characteristic of a sample must be discrete values or must be discretized in a stage prior to the generation of the tree.

The operation of the algorithm follows a recursive process, where if after performing divisions based on the gain of the information all the methods belong to the same class, then a leaf node is generated. Otherwise, the information gain for each attribute is calculated and new decision nodes are generated with the attribute with the highest information gain (Equation 5).


(5) $$E\,n\,t\,r\,o\,p\,y\,\left(S\right)=-\sum_{i=1}^{m}p_{i}\,l\,o\,g_{2}\,p_{i}$$

where:


m=number⁢of⁢classes,

$$p_{i}=\mathrm{proportion}\,\mathrm{of}\,\mathrm{examples}\,\mathrm{in}\,S\,\mathrm{that}\,\mathrm{belong}\,\mathrm{to}\,\mathrm{class}\,i.$$

*Logistic model trees*: Although decision trees are usually generated in a hierarchical way and perform divisions using metrics such as entropy or information gain, there are some models, like logistic model trees (LMTs) (Landwehr et al., 2005), that perform logistic regressions mixed with decision trees to classify the different data. Specifically, LMTs follow a similar tree-structure that divides into leaves and decision nodes, like C4.5. The main difference is that, while in C4.5 each leaf node represents the class label that corresponds to a piece of data, in LMTs, each leaf node is a logistic regression.

The division of the tree into decision nodes and leaf nodes is performed with the LogitBoost algorithm (Friedman et al., 2000), so that a root node is generated that divides the set into two groups. Each subgroup is divided again with the same algorithm until the divisions are only leaf nodes or a stop criterion is reached, such as the information gain being less than a certain value. At each split node is where a logistic regression is performed to determine the path for the tree to follow (Equation 6).


(6) $$\hat{y}\,\left(x\right)=\sigma \,\left(\beta_{0}^{\left(l\right)}+\sum_{j=1}^{p}\beta_{j}^{\left(l\right)}\,x_{j}\right)$$

where:


l=index⁢of⁢the⁢leaf⁢where⁢x⁢falls⁢in⁢the⁢tree,

$$\beta_{j}^{\left(l\right)}=\mathrm{coefficients}\,\mathrm{of}\,\mathrm{the}\,\mathrm{logistic}\,\mathrm{model}\,\mathrm{in}\,\mathrm{leaf}\,l,$$

$$\sigma \,\left(z\right)=\frac{1}{1+e^{-z}}=\mathrm{sigmoid}\,\mathrm{function},$$

p=number⁢of⁢predicto⁢rs.

In binary classification, class 1 is predicted if $\hat{y}\,\left(x\right)=0.5;$ otherwise, class 0 is predicted.

#### 2.2.1 Description of dataset partitions

The eye-tracking task was carried out with 93 participants (Table 1), and for each participant four datasets were obtained: “full variables” (158), “basic variables” (24), “prior variables” (60), and “secondary variables” (72) (see Procedure: Task Performance). For each of these datasets and to analyze the performance of the classification algorithms, a partitioning of the data was performed, using a stratified five-folds methodology. With this methodology, each algorithm trains with a small subset of the data (four-fold) and tries to predict data that it has not seen in the training phase with another subset of the data (one-fold). To summarize the performance of the different algorithms, the F1-Score (Van Rijsbergen, 1979) metric is used because it is a commonly used metric in ML that incorporates “precision” (Manning et al., 2008) and “recall” (Manning et al., 2008) metrics in its calculation. F1-Scores range from 0 to 1, where 0.0–0.5 is poor performance (low precision and/or recall); 0.5–0.7 is moderate performance (model is improving but not optimal); 0.8–0.9 is good performance (with some room for improvement); and 0.9–1.0 is excellent performance (nearly perfect precision and recall).

As shown in Equation 7, precision and recall were defined as follows:


(7) $$P\,r\,e\,c\,i\,s\,i\,o\,n=\frac{T\,P}{T\,P+F\,P};R\,e\,c\,a\,l\,l=\frac{T\,P}{T\,P+F\,N}$$

where:

TPs (true positives) represent the number of elements of the positive class correctly classified by the model.

FPs (false positives) represent the number of elements of the negative class classified as positive class by the model.

FNs (false negatives) represent the number of elements of the positive class classified as negative class by the model.

An F1-Score is the weighted average of precision and recall, and it takes both FPs and FNs into account. This metric is calculated, following (Equation 8):


(8) $$F\,1-S\,c\,o\,r\,e=2\cdot \frac{P\,r\,e\,c\,i\,s\,i\,o\,n\cdot R\,e\,c\,a\,l\,l}{P\,r\,e\,c\,i\,s\,i\,\text{ó}\,n+R\,e\,c\,a\,l\,l}$$

Additionally, to the 5-fold partitions, the above-mentioned datasets were divided into five different scenarios in which different between group comparisons were tested (TD–ASD–DLD, TD–ASD, TD–DLD, ASD–DLD, TD–Disorder). To achieve this, a stepwise analysis of the results was required, comparing the various group combinations across the four datasets and applying the six selected algorithms.

## 3 Results

To achieve specific objectives, each comparison of groups will be described in a separate subsection.

### 3.1 Split for comparison between the TD, ASD, and DLD groups

The aim of this first test was to study the behavior of the different algorithms with four datasets in the classification of the three groups of participants. The F1-Score results are shown in Table 2.

**TABLE 2:** F1-Score for the TD, ASD, and DLD groups per the four datasets across algorithms.

Five-folds	Full variables	Basic variables	Prior variables	Secondary variables	Mean
Naive Bayes	0.712	0.695	0.724	0.613	0.686
Ripper	0.553	0.614	0.590	0.579	0.584
One rule	0.543	0.622	0.511	0.533	0.552
PART	0.511	0.586	0.552	0.541	0.548
C4.5 (J48)	0.591	0.591	0.600	0.564	0.587
LMT	0.715	0.716	0.667	0.624	0.681
Mean	0.604	0.637	0.607	0.576	0.606
MAX	0.715	0.716	0.724	0.624	

F1-Scores ranged from 0 to 1, where 0.0–0.5 was a poor performance; 0.5–0.7 was a moderate performance; 0.7–0.9 was a good performance; and 0.9–1.0 was an excellent performance.

Table 2 shows values above 0.7 in some cases. The best algorithms (above 0.7) were for LMTs with full and basic variables, together with the Naive Bayes algorithm for full and prior variables. The best overall result was achieved with the Naive Bayes algorithm with the prior variables (0.724).

### 3.2 Split for comparison between the TD and ASD groups

The aim of this second test was to study the behavior of the different algorithms in the classification of the TD group versus the ASD group of participants. The results appear in Table 3.

**TABLE 3:** F1-Score for the TD and ASD groups per the four datasets across algorithms.

Five-folds	Full variables	Basic variables	Prior variables	Secondary variables	Mean
Naive Bayes	0.825	0.813	0.798	0.767	0.801
Ripper	0.690	0.839	0.690	0.789	0.752
One rule	0.683	0.811	0.796	0.719	0.752
PART	0.742	0.833	0.716	0.808	0.775
C4.5 (J48)	0.759	0.849	0.682	0.808	0.775
LMT	0.822	0.867	0.749	0.840	0.820
Mean	0.754	0.835	0.739	0.789	0.779
MAX	0.825	0.867	0.798	0.840	

F1-Scores ranged from 0 to 1, where 0.0–0.5 was a poor performance; 0.5–0.7 was a moderate performance; 0.7–0.9 was a good performance; and 0.9–1.0 was an excellent performance.

Table 3 shows values above 0.8 in most cases, except for the prior variables. On average, the best-performing algorithms were Naive Bayes and LMTs, and the best dataset was basic variables. The best overall result for the dataset and algorithm combination was obtained for the LMT algorithm with the basic variables (0.867).

### 3.3 Split for comparison between the TD and DLD groups

The aim of the third test was to study the behavior of the different algorithms in the classification of the TD group versus the DLD group. The results are shown in Table 4.

**TABLE 4:** F1-Score for the TD and DLD groups per the four datasets across algorithms.

Five-folds	Full variables	Basic variables	Prior variables	Secondary variables	Mean
Naive Bayes	0.912	0.824	0.854	0.871	0.865
Ripper	0.824	0.840	0.838	0.824	0.832
One rule	0.813	0.728	0.806	0.852	0.800
PART	0.806	0.840	0.785	0.840	0.818
C4.5 (J48)	0.803	0.882	0.812	0.809	0.827
LMT	0.812	0.912	0.855	0.856	0.859
Mean	0.828	0.838	0.825	0.842	0.833
MAX	0.912	0.912	0.855	0.871	

F1-Scores ranged from 0 to 1, where 0.0–0.5 was a poor performance; 0.5–0.7 was a moderate performance; 0.7–0.9 was a good performance; and 0.9–1.0 was an excellent performance.

Table 4 shows F1-Score values above 0.8 in all cases except one, even reaching values above 0.9. The overall best-performing algorithms were Naive Bayes for the full set of variables and LMTs for the basic variables, both reaching a value of 0.912.

### 3.4 Split for comparison between the ASD and DLD groups

The aim of this fourth test was to study the behavior of the different algorithms in the classification of the two types of disorders. The results are shown in Table 5.

**TABLE 5:** F1-Score for the ASD and DLD groups per the four datasets across algorithms.

Five-folds	Full variables	Basic variables	Prior variables	Secondary variables	Mean
Naive Bayes	0.626	0.632	0.609	0.529	0.599
Ripper	0.508	0.611	0.427	0.506	0.513
One rule	0.444	0.522	0.408	0.540	0.479
PART	0.446	0.510	0.530	0.465	0.488
C4.5 (J48)	0.408	0.509	0.408	0.442	0.442
LMT	0.531	0.509	0.531	0.460	0.508
Mean	0.494	0.549	0.486	0.490	0.606
MAX	0.626	0.632	0.609	0.540	

F1-Scores ranged from 0 to 1, where 0.0–0.5 was a poor performance; 0.5–0.7 was a moderate performance; 0.7–0.9 was a good performance; and 0.9–1.0 was an excellent performance.

In this case, the results were approximately 0.5, with some of them around 0.6. Again, the best value of all was obtained with the Naive Bayes algorithm, using the basic variables (0.632).

### 3.5 Split for comparison between the TD and disorder groups (ASD and DLD)

The aim of the fifth test was to study the behavior of the different algorithms in the classification between the TD and disorder groups. The disorder group was the union of the ASD and DLD participants. The results are shown in Table 6.

**TABLE 6:** F1-Score for the TD and Disorder Groups (ASD and DLD) per the four datasets across algorithms.

Five-folds	Full variables	Basic variables	Prior variables	Secondary variables	Mean
Naive Bayes	0.860	0.828	0.850	0.817	0.839
Ripper	0.785	0.807	0.771	0.785	0.787
One rule	0.697	0.828	0.697	0.750	0.743
PART	0.816	0.839	0.762	0.828	0.811
C4.5 (J48)	0.795	0.838	0.709	0.828	0.793
LMT	0.870	0.881	0.870	0.827	0.862
Mean	0.804	0.837	0.777	0.806	0.806
MAX	0.870	0.881	0.870	0.828	

F1-Scores ranged from 0 to 1, where 0.0–0.5 was a poor performance; 0.5–0.7 was a moderate performance; 0.7–0.9 was a good performance; and 0.9–1.0 was an excellent performance.

Table 6 shows values close to 0.9 in most cases. The LMT algorithm performed best across all datasets, particularly with the basic variables, which yielded the highest value (0.881).

### 3.6 Selection of the best algorithms and datasets for the compared groups

In summary, Table 7 shows the results of the algorithm and dataset combinations that achieved the highest F1-Score values for each comparison group.

**TABLE 7:** Summary of findings.

Comparison groups	Best dataset	Best algorithm	F1-Score
TD vs. ASD vs. DLD	Prior	Naive Bayes	0.724
TD vs. ASD	Basic	LMT	0.867
TD vs. DLD	Full/basic	Naive Bayes/LMT	0.912
ASD vs. DLD	Basic	Naive Bayes	0.632
TD vs. Disorders (ASD + DLD)	Basic	LMT	0.881

F1-Scores ranged from 0 to 1, where 0.0–0.5 was a poor performance; 0.5–0.7 was a moderate performance; 0.7–0.9 was a good performance; and 0.9–1.0 was an excellent performance.

In Table 7, in four out of five comparisons, the best results were obtained with the basic variables dataset, and the LMT and Naive Bayes algorithms achieved the same top results. Moreover, the highest model accuracy was achieved when comparing the TD versus DLD groups, although good accuracy was also obtained when comparing the TD versus Disorder groups (ASD + DLD), as well as TD versus ASD.

### 3.7 Model

**FIGURE 3:**
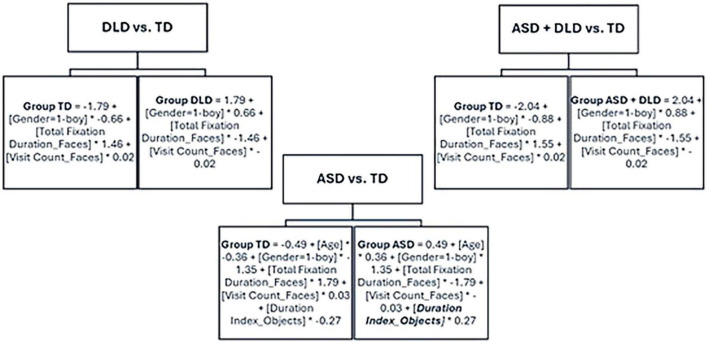
Model obtained for the different group combinations, using the full variables dataset and the LMT algorithm.

The main objective of this study was to explore and select the most efficient XML algorithms to distinguish between TD, ASD, and DLD samples, using eye-tracking data, to assist clinicians in making decisions. Moreover, to analyze if XAI algorithms can help clinicians explain the specific characteristics for each group, we studied the model resulting from the learning of this algorithm. The model obtained for the different group combinations using the LMT algorithm through the full-variable dataset is presented in Figure 3.

Analyzing the model obtained with the DLD versus the TD groups, we observed that it was a simple model in which, first, we obtained a very simple tree with a single leaf node. Second, in the leaf node we obtained a function in which only the variables “gender,” “total fixation duration faces,” and “visit count faces” were considered, which indicates that these were the most important variables that would be the first examined. The same result with the same variables was found in the comparison of the two disorder groups (ASD + DLD) versus TD, unlike the model obtained with the ASD versus the TD groups, which included an additional eye-tracking variable specific to this comparison: “duration index objects.”

## 4 Discussion

Here we analyze our conclusions while considering the specific objectives of this study. Our first objective was determining which dataset was the most useful for generating explainable classification models, and the second was to identify which dataset better differentiated participants based on their diagnosis. The best classification results were achieved with the basic variables dataset, which included all metrics related to the AOIs of total faces and total objects, comprising only 24 of the 156 possible variables. Thus, increasing the number of variables in the ML model by including parameters from smaller AOIs, such as the mouth and eyes, or more specific AOIs, such as emotion or object interests, did not improve the group classification results. Furthermore, our results suggest that the essential features to be included in the dataset were eye-tracking variables that captured differences between objects and faces. This finding aligns with previous studies, where attention to faces versus non-social stimuli is identified as a distinguishing characteristic of ASD (Anderson et al., 2006; Pierce et al., 2011; Vacas et al., 2021c, 2022a). On the other hand, although previous studies have found differences in the exploration of facial regions (Jónsdóttir et al., 2023) and facial emotional expressions (Polzer et al., 2024; Vacas et al., 2022b) between ASD and TD, these variables do not appear to be critical for the classification accuracy achieved by the different algorithms.

To evaluate the performance of the obtained ML models, comparisons were made between the different participant groups. The results reflect good accuracy in distinguishing the disorder groups (ASD and DLD) from the TD group, both individually and combined. However, the accuracy was less satisfactory when comparing the two disorder groups with each other (ASD vs. DLD). These findings are consistent with the overlap in deficits observed between both disorders (Félix et al., 2024). Furthermore, given the limited number of studies comparing these NDDs in terms of ocular behavior during social attention tasks (Vacas et al., 2024), the results provide valuable insights into shared characteristics of both disorders and confirm the challenges of making a differential diagnosis at early ages. In conclusion, the eye-tracking variables obtained from preference tasks (specifically the basic variables related to object and face AOIs), combined with ML algorithms, were effective in distinguishing disorder cases from TD participants, although they did not differentiate between ASD and DLDs. Perhaps the findings reported in previous studies, using ML and eye-tracking data to classify ASD and TD groups (Kollias et al., 2021), might have overestimated their accuracy because they only included comparisons between ASD and TD groups, avoiding comparisons with groups that share similar symptomatology.

Conversely, regarding our objective of analyzing which ML model was more efficient in distinguishing between TD, ASD, and DLD samples using eye-tracking data, both the LMT algorithm and Naive Bayes performed significantly better in the various tests conducted. Additionally, our objective included ensuring that the information provided by the XLM algorithms could be interpreted to explain the disorders and assist in decision-making in clinical practice. In this regard, the analysis of the model generated by the LMT algorithm (Figure 3) in the comparison of the different groups provided more specific results than those obtained by comparing different datasets. The XLM algorithms allow access to information on the eye-tracking variables that determine case classification among the various groups. The models generated by the LMT algorithm showed that, to differentiate the ASD and DLD groups from the TD group, the relevant eye-tracking variables were Total Fixation Duration to Faces and Visit Count to Faces (both lower in the disorder groups). Additionally, when comparing the ASD group to the TD group, the specific variable added to the previous variables was Duration Index to Objects. Perhaps both disorders share common aspects regarding attention to faces —less total fixation time and fewer visits to faces compared to their TD counterparts. However, longer fixation duration on objects was a distinctive marker of the ASD group, though it did not emerge as a relevant variable in the ML models for the DLD or TD groups. Longer fixation duration can be interpreted, from the perspective of attentional processes, as a difficulty in disengaging attention from objects. Previous studies had already indicated this as a characteristic of ASD that may be a contributing factor to some of the core features of autism, such as social interaction difficulties (Landry and Bryson, 2004).

These results suggest important directions for future work, including incorporate additional XML algorithms, such as Gradient Boosting Trees and Explainable Boosting Machines (EBMs). These algorithms represent a significant advancement in the field of interpretable machine learning, as they combine strong predictive performance with accessible interpretability. In the next phase of our research, we also intend to expand our analytical framework by integrating multimodal data, combining eye-tracking variables with additional behavioral, cognitive, or neurophysiological measures. This integration could enhance both the robustness and the clinical applicability of our models, contributing to the development of effective and interpretable tools for the differential diagnosis of neurodevelopmental disorders.

From this study, we recommend including different diagnostic groups when applying ML algorithms to classify diagnostic groups to avoid an overestimation bias in classification accuracy. Additionally, the use of XLM algorithms is recommended, as they allow access to information about the relevant variables that explain the differences between groups and can aid in explaining the disorder

One of the distinctive aspects of this study is the use of data obtained through tasks specifically designed to capture attentional patterns characteristic of children with autism. This methodological decision aligns with the goal of advancing toward differential diagnosis through the application of XML techniques. The quality and specificity of the input data are key factors for both the performance and interpretability of XML models.

This study had limitations, particularly regarding the scope of the experiment and the sample size. Additionally, our diagnostic tools could have been supplemented with further assessments. However, ours were the only tools available at the time of the research. Therefore, it is crucial to replicate this study, using the most current behavioral assessments to ensure that future research provides more accurate and up-to-date data. Moreover, diagnoses at early ages are provisional and may evolve over time. Consequently, our findings should be interpreted with caution.

## 5 Conclusion

Our findings are multifaceted. The accuracy achieved in distinguishing participants with ASD and DLD from TD participants was excellent to very good, while it was moderate for differentiating between ASD and DLD, suggesting that the approach is more effective as a screening tool than for differential diagnosis. Notably, the most informative eye-tracking variables were those that differentiated between social and non-social stimuli in general; including additional variables with specific details of the stimuli did not enhance accuracy. Furthermore, Naive Bayes and LMT algorithms yielded models better tailored to our data and objectives. Additionally, this study uncovered specific values for key indices that may help identify distinct markers (e.g., Duration Index to Objects) for each condition, moving closer to the development of new tools that support early differential diagnoses. Overall, ML has proven its utility in processing a large amount of data generated from eye movement recordings and appears essential for implementing intelligent systems. These systems can serve in preliminary steps toward the implementation of computer-aided diagnoses for NDDs like ASD and DLDs.

## Data Availability

The raw data supporting the conclusions of this article will be made available by the authors, without undue reservation.

## Footnotes

1
https://pixabay.com/es/
